# The establishment of new protein expression system using N starvation inducible promoters in *Chlorella*

**DOI:** 10.1038/s41598-020-69620-9

**Published:** 2020-07-29

**Authors:** Jun-Hye Shin, Juyoung Choi, Jeongmin Jeon, Manu Kumar, Juhyeon Lee, Won-Joong Jeong, Seong-Ryong Kim

**Affiliations:** 1 Department of Life Science, Sogang University, Seoul, South Korea; 2 Korea Research Institute of Bioscience and Biotechnology, Daejeon, South Korea

**Keywords:** Biotechnology, Microbiology, Plant sciences

## Abstract

*Chlorella* is a unicellular green microalga that has been used in fields such as bioenergy production and food supplementation. In this study, two promoters of N (nitrogen) deficiency-inducible *C*hlorella *v*ulgaris *N D*eficiency *I*nducible (*CvNDI*) genes were isolated from *Chlorella vulgaris* UTEX 395. These promoters were used for the production of a recombinant protein, human granulocyte-colony stimulating factor (hG-CSF) in *Chlorella vulgaris* UTEX 395 and *Chlorella* sp*.* ArM0029B. To efficiently secrete the hG-CSF, the protein expression vectors incorporated novel signal peptides obtained from a secretomics analysis of *Chlorella* spp. After a stable transformation of those vectors with a codon-optimized *hG-CSF* sequence, hG-CSF polypeptides were successfully produced in the spent media of the transgenic *Chlorella*. To our knowledge, this is the first report of recombinant protein expression using endogenous gene components of *Chlorella*.

## Introduction

*Chlorella* is a single-celled eukaryote belonging to the green algae. It can grow phototrophically, heterotrophically, or mixotrophically to high biomass, producing different amounts of proteins and lipids^1^. It grows rapidly, dividing into four cells in about 6 h. Because *Chlorella* synthesizes large amounts of proteins and lipids, it has been used as a raw material for biodiesel and also as a food additive^2–4^. *Chlorella* undergoes posttranslational modifications such as protein N-glycosylation^5^. The use of *Chlorella* for the production of valuable proteins has therefore been attempted for more than two decades^6–9^. Chen et al.^10^ showed expression of rabbit neutrophil peptide using a translational enhancer omega in *Chlorella ellipsoidea.* Kim et al.^11^ produced flounder growth hormone using a 35S cauliflower mosaic virus (35S) promoter in *C. ellipsoidea* and showed a 25% growth increase of flounder fry. No recombinant proteins produced from *Chlorella* species have yet been commercialized, although the species has been studied intensively for the potential production of biodiesel. An optimized expression system for producing proteins in *Chlorella* is urgently needed.

Recombinant protein production studies require a highly efficient method for the transformation of *Chlorella*. Various transformation techniques, including electroporation, PEG transformation, particle bombardment, and *Agrobacterium* cocultivation, have been used for the efficient transformation of *Chlorella* spp*.*^10–14^. Recently, Kumar et al.^15^ reported an effective method for enhancing transformation efficiency by more than 100-fold using electroporation combined with efficient protoplasting of *Chlorella*.

Besides efficient transformation, an optimized system for the endogenous expression of proteins is indispensable. An expression system involving appropriate promoters is one of the most important factors for high-efficiency production of recombinant proteins. In *Chlamydomonas reinhardtii*, the promoter of the heat shock protein gene *HSP70a* has been widely used for the expression of foreign genes in *C. reinhardtii*^16,17^. In recent years, the promoter of the nitrate reductase gene has been used for recombinant protein production in *Phaeodactylum tricornutum*^18,19^. The addition of NaNO_3_ to growth media caused an increase in the activity of the nitrate reductase promoter, resulting in an increase in production of recombinant protein. It has been reported that both the cauliflower mosaic virus 35S promoter and the *C. reinhardtii HSP70* promoter are effective in transgenic *C. vulgaris*^15^. Recently, the Photosystem I protein D (*psaD*) gene homologous to *C. reinhardtii psaD* was identified in *C. vulgaris in silico*^20^*.* The 0.4 kb *psaD* promoter region was shown to act in *Nicotiana benthamiana* as well as in *C. reinhardtii*, but not in *Chlorella*, although it was not as strong as the promoter of the *C. reinhardtii psaD* gene^20^. To date, the use of the promoters of the endogenous genes of *Chlorella* has not been studied in *Chlorella*.

Under N depletion conditions, *Chlorella* stores carbon as triglycerides and starch, causing massive lipid production in the cell^21^. Recently, it has been reported that protein production under these conditions depends upon the N level and the duration of treatment^22^. The study showed that protein productivity in N starvation media was more than 40% higher than that in N-sufficient media over 4 days of *Chlorella* culture. Moreover, lipid productivity under the N starvation media was least at the fourth day of incubation, indicating that N starvation could be used for enhancing recombinant protein production.

Granulocyte-colony stimulating factor (G-CSF) is a member of CSF family: hormone-like glycoproteins produced by a variety of tissues to regulate the proliferation and differentiation of neutrophilic granulocytes^23–25^. G-CSF functions as a primary regulatory factor controlling the neutrophil response to inflammatory stimuli^24^. Cloning and expression of a human G-CSF (*hG-CSF*) gene in *Escherichia coli* was achieved in 1986 by Amgen^26^. The commercialized hG-CSF, which functions as an immunostimulator, has been used for patients with congenital or acquired neutropenia, and forms of recombinant hG-CSF being sold include filgrastim, pegfilgrastim, and lenograstim^25^. The nonglycosylated form was reported to have similar efficacy to that of the O-glycosylated form, indicating that the glycosylation of hG-CSF might not be important for the function^27^.

Recently, a new *Chlorella* species, ArM0029B, was isolated from drift ice in the Arctic region. The organism was characterized, and its genome was partially sequenced^28,29^. ArM0029B grows rapidly and accumulates high levels of lipids at a range of temperatures^28^. ArM0029B might be more easily handled and more productive than other *Chlorella* species.

In this study, we developed a novel expression system controlled by endogenous gene promoters to produce desired proteins in *Chlorella*. Two genes induced by N deficiency were identified using RNA-Seq of *Chlorella* ArM0029B grown under N deficiency. The homologous genes were isolated from *C. vulgaris* UTEX 395 using BLAST searching. The genes were named *C*hlorella *v*ulgaris *N D*eficiency *I*nducible *1* (*CvNDI1*) and *2* (*CvNDI2*). The promoter regions of those genes were used to construct expression vectors for the *hG-CSF* gene. Novel signal peptides (SPs) were also used for the production of target proteins in the culture media. The vector systems were successfully used to produce hG-CSF in transgenic *Chlorella* grown under media lacking N.

## Results

### Screening of N deficiency-induced *CvNDI1 and CvNDI2* genes from *Chlorella* UTEX 395

**Figure 1:**
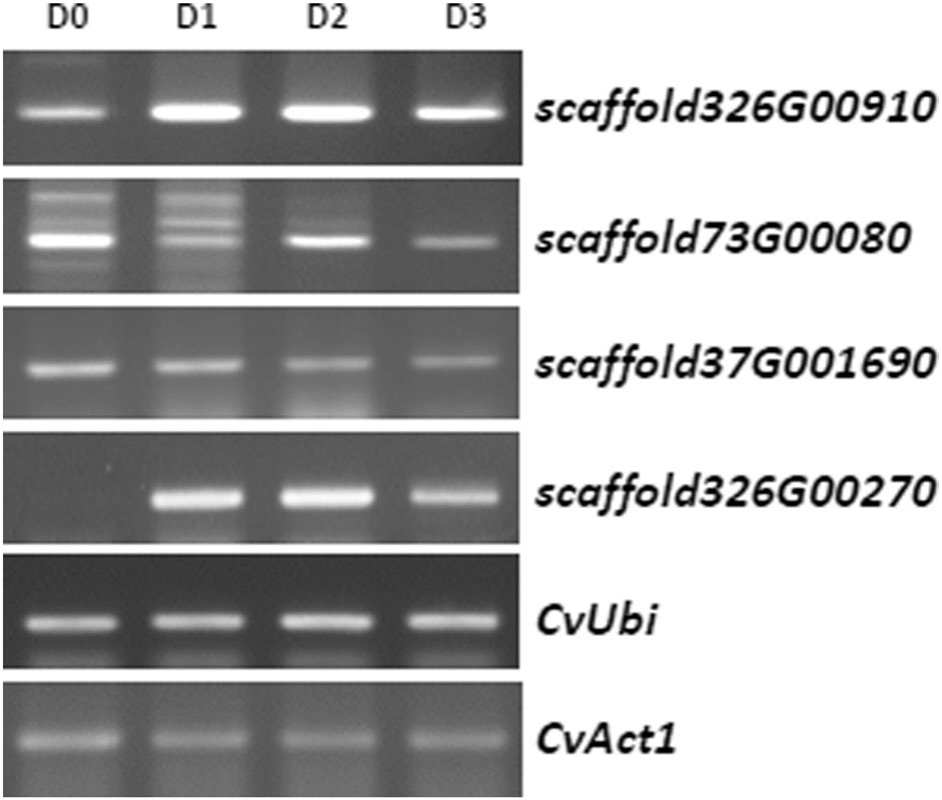
Expression pattern of N starvation-induced genes screened from UTEX395 based on RNA-Seq data from ArM0029B. (A) *Chlorella* UTEX 395 grown in N-rich media was transferred into N-deficient media and then cultivated for 3 days. Total RNA was extracted from the cells harvested every day after the transfer, and RT-PCR was performed using scaffold-specific primers. (D0) before transfer; (D1) 1 day after transfer; (D2) 2 days after transfer; (D3) 3 days after transfer. Both *CvUbi* and *CvAct1* were used for normalization.

The development of an optimal promoter is a prerequisite for the production of recombinant proteins in heterologous biological systems. For the controlled expression of recombinant proteins in *Chlorella*, inducible promoters have been investigated as potential target promoters. N deprivation is well known to produce a dramatic increase in lipid content in *C. vulgaris*^30^^,^ and these lipids can be used in biodiesel production. To identify the genes induced under N starvation, an arctic *Chlorella* sp., ArM0029B^28^^,^ was subjected to RNA sequencing under N-deficient conditions. Differential expression (DE) analysis using the RNA-seq data identified 20 genes whose expression was increased more than tenfold and were thus considered significant (Supplementary Table 1). The transcript level of *scaffold326G00910* was increased up to 20 times. Three genes, *scaffold326G00270*, *73G00080*, and *253G00910*, also showed significant increases in transcription levels (Supplementary Table 1). Using these four gene sequences, BLAST search was performed against the UTEX 395 sequencing data, and the four corresponding candidate homologous genes were isolated from the genome sequence of UTEX 395. To confirm the expression of those genes in the UTEX 395 under N-deficient conditions and further screen for an optimal gene among the candidates, RT-PCR was performed after 3 days cultivation of UTEX 395 in media lacking N. The numbers of transcripts of the *scaffold326G00910* and *326G00270* homologs were shown to be significantly elevated under these conditions, consistent with the results of the DE analysis (Fig. 1). The expression of the *scaffold326G00910* homolog was rapidly induced on day 1 in the N-deficient media, expression was maintained until day 2, and then, the expression level decreased to about half on day 3 (Fig. 1). The *scaffold326G00270* homolog showed a similar expression pattern to that of the *scaffold 326G00910* homolog, although the transcript of the *scaffold326G00270* homolog was almost undetectable in normal growth media containing N, indicating that its expression is N starvation specific (Fig. 1). The other genes examined (*scaffold37G001690* and *73G00080* homologs) were not induced by N starvation in UTEX395 (Fig. 1). To examine the rate at which the transcription of the *scaffold326G00910* and *326G00270* homologs was triggered under N starvation, the expression patterns of the two genes were further checked by performing quantitative PCR (qPCR) of the genes at 0, 6, 12, and 24 h (N0, N6, N12, and N24, respectively) after N starvation treatment (Supplementary Fig. 1). Transcription of both the *scaffold326G00910* and *326G00270* homologs was rapidly triggered in 6 h to about 1,500-fold and 30-fold, respectively. The transcript levels at N12 increased by up to 3,500-fold and 60-fold, respectively, compared with N0. The results indicate that the *scaffold326G00910 and 326G00270* homologs are expressed quickly and strongly under N starvation conditions. Therefore, the *scaffold326G00910 and 326G00270* homologs were selected for the development of a N starvation deficiency-inducible promoter gene expression system and were named *CvNDI1* and *CvNDI2*, respectively (GenBank accession numbers MN971585 and MN971586).

The amino acid sequences of the *CvNDI1* and *CvNDI2* genes were deduced from the full-length DNA sequences obtained by BLAST search. BLAST searches using the amino acid sequences showed that CvNDI1 has a conserved urea carboxylase domain (Supplementary Fig. 2). CvNDI1 showed 79% homology with the urea carboxylase of *Micractinium conductrix* and 77% homology with that of *C. sorokiniana* (Supplementary Fig. 2). The biotin carboxylation domain is conserved in the N-terminal region of CvNDI1, and the carboxyltransferase domain is conserved at the C-terminal (blue and red boxes, respectively, in Supplementary Fig. 2). Urea carboxylase converts urea into ammonium, the first step for the utilization of urea as a N source^31,32^. Urea generated by the degradation of N compounds is known to be used as a N source in many plants, fungi, and bacteria^33–35^.

**Figure 2:**
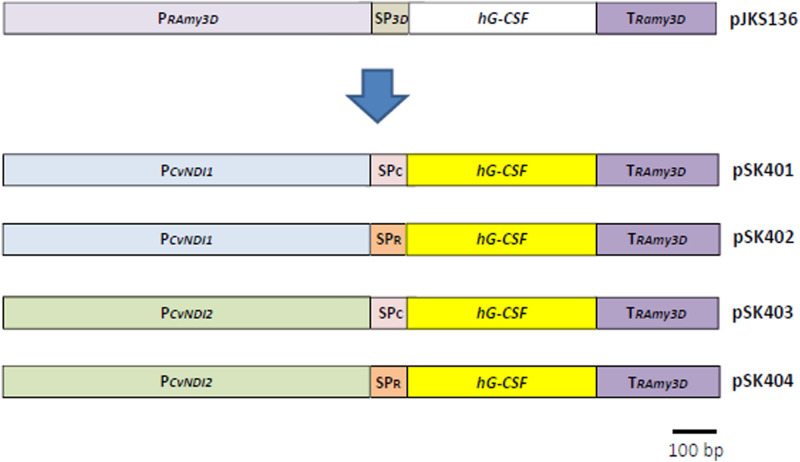
Construction of *hG-CSF* expression vectors controlled by *CvNDI* promoters. Using pJKS136 as a backbone, the *RAmy3D* promoter (P*RAmy3D*) was replaced with either *CvNDI1* (pSK401 and pSK402) or *CvNDI2* (pSK403 and pSK404). The *hG-CSF* sequence of pJKS136 (white box) was replaced with a codon-optimized *hG-CSF* sequence (yellow box) for *Chlorella.* The signal peptide of a putative cellulase (SP_c_), screened from our secretome data from UTEX395, was introduced into pSK401 and pSK403 for the transformation of UTEX 395. The signal peptide of a Ras-related RABF1 (SP_R_), screened from our secretome data of ArM0029B, was introduced into pSK402 and pSK404 for the transformation of ArM0029B. The black bar indicates a length of 100 bp.

The CvNDI2 sequence showed homologies with the ammonium transporters (AMTs) of *M. conductrix* and *C. sorokiniana* of 73% and 74%, respectively (Supplementary Fig. 3a). Hydrophobicity analysis indicated that the CvNDI2 protein contains 11 transmembrane domains, which are generally conserved among the AMT proteins of a range of organisms (Supplementary Fig. 3b). The AMT plays an important role in N metabolism by maintaining an optimal level of N in the cell^36,37^. Therefore, both *CvNDI1* and *CvNDI2* genes are expected to be involved in metabolism to support N sources for cell survival under N starvation conditions.

**Figure 3:**
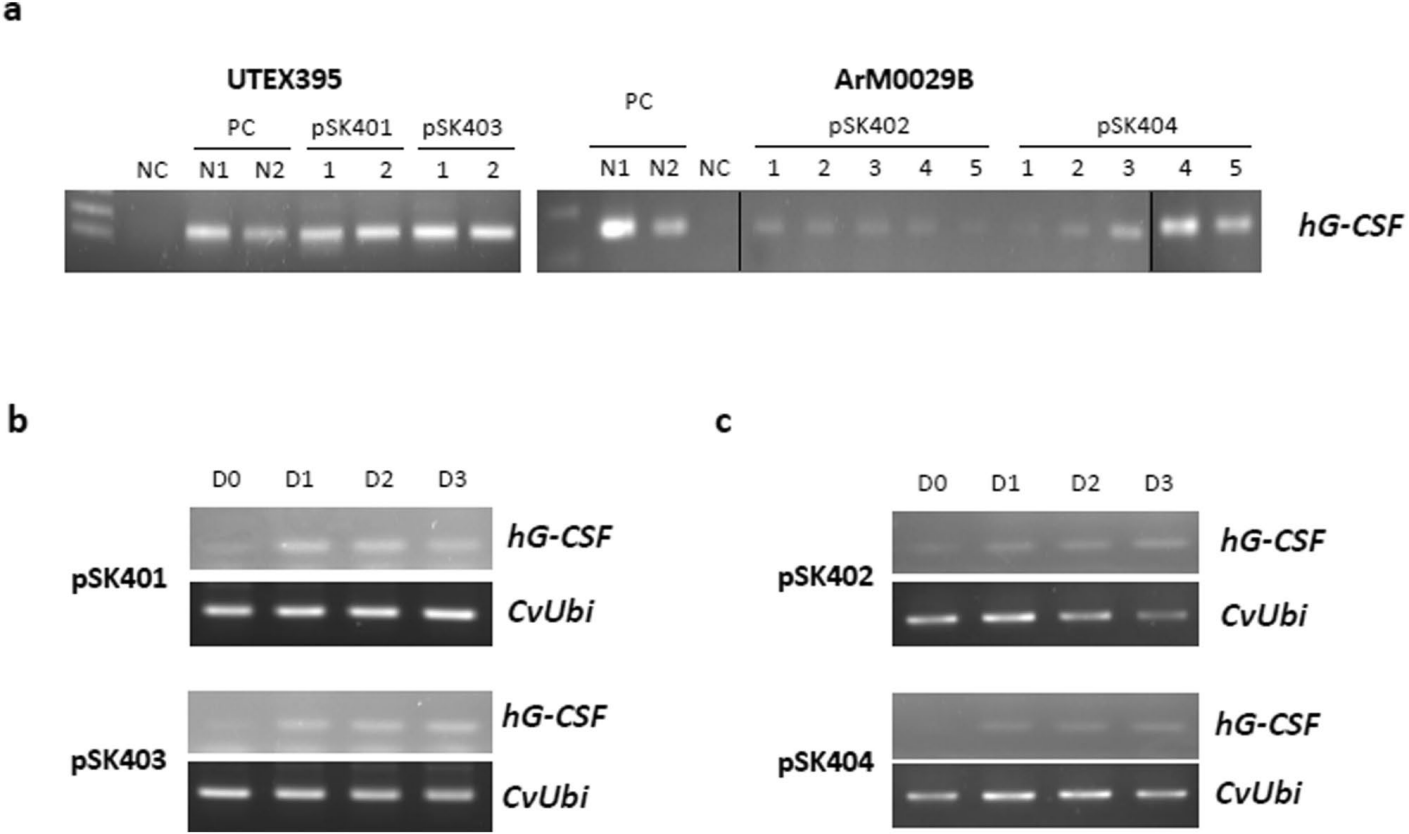
Molecular characterization of transgenic *Chlorella* spp. (**a**) Genomic DNA PCR of the transgenic *Chlorella,* UTEX 395 (left panel) and ArM0029B (right panel), using *hG-CSF* primers. NC, negative control; PC, positive control. Vertical black lines indicate the grouping of gels cropped from different parts of the same gel. (**b**) Expression of an *hG-CSF* transcript by N deficiency in the transgenic UTEX 395. (**c**) Expression of *hG-CSF* transcript by N deficiency in the transgenic ArM0029B. Transgenic *Chlorella* grown in standard media was transferred to N-deficient media and harvested at 0 days (D0), 2 days (D2), and 3 days (D3) after the transfer. *CvUbi* gene was used for normalization.

### Construction of *hG-CSF* expression vectors driven by *CvNDI* promoters

To construct protein expression vectors driven by the promoters of the *CvNDI* genes, the sequences of the promoter region spanning a 1 kb-long region upstream of the translation start codon (ATG), including the 5′-untranslated region (UTR), were amplified by PCR, using the genomic DNA of UTEX 395 as templates (Supplementary Fig. 4). As shown in Fig. 2, the promoter sequences of the *CvNDI1* and *CvNDI2* genes were inserted into pJKS136, replacing the *RAmy3D* promoters.

**Figure 4:**
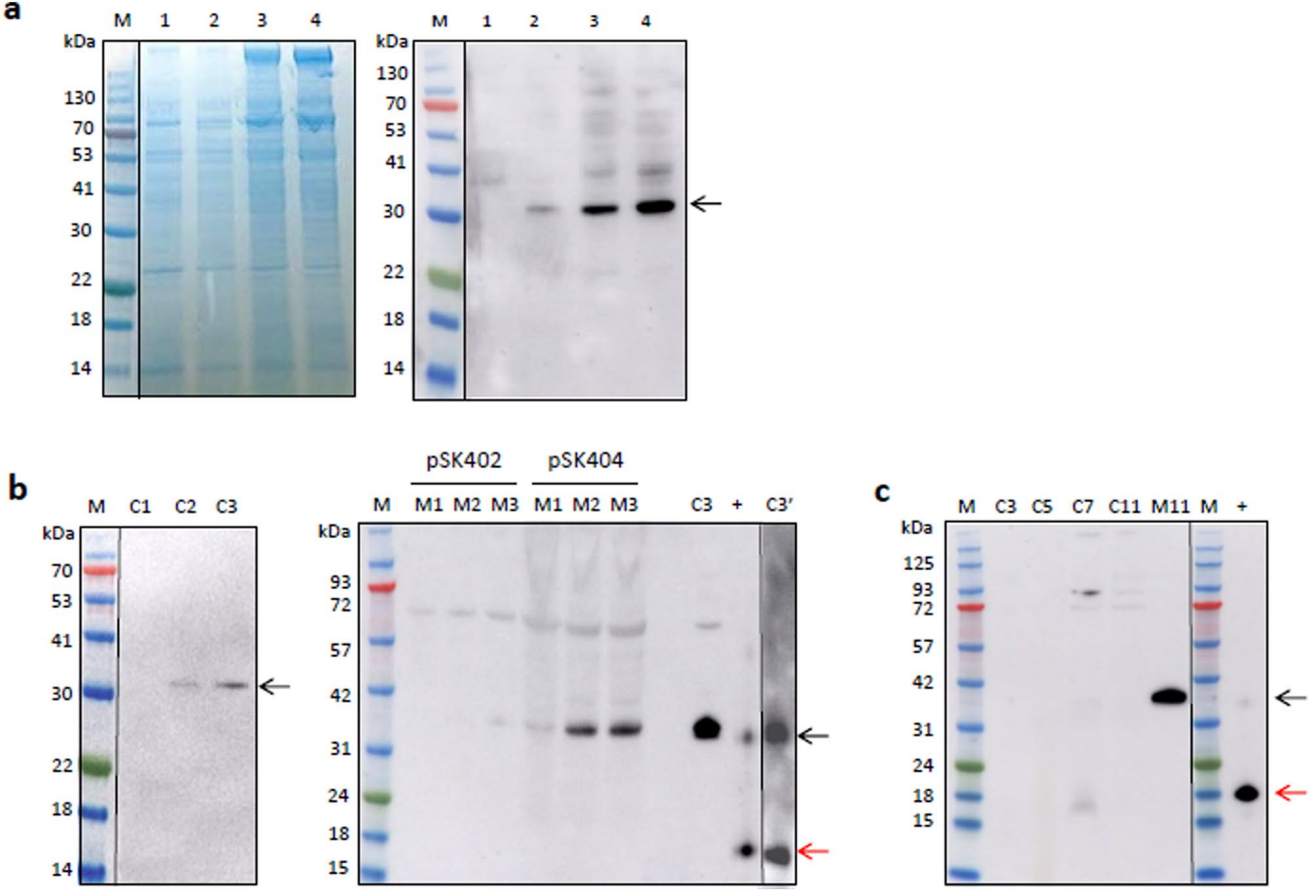
Expression of hG-CSF polypeptides in the transgenic *Chlorella* spp. (**a**) Transgenic *Chlorella* cells grown in standard N-rich media were transferred to N-deficient media and harvested the following day. Total proteins from the cell lysates were separated on 12% NuPAGE bis–tris gel (left panel) and transferred to nitrocellulose membrane for western blotting (right panel) using a polyclonal antibody of hG-CSF diluted 1: 2000. M, protein size marker; (1) UTEX 395-pSK401; (2) UTEX 395-pSK403; (3) ArM0029B-pSK402; (4) ArM0029B-pSK404. (**b**) Total proteins of ArM0029B-pSK404 were extracted from either cell lysates (left panel) or spent media (right panel) for 3 days after transfer to the N-deficient media and used for western blotting. The media was concentrated 250-fold and used for the western blotting. M, protein size marker; C1, protein extracts of cell lysates from day 1; C2, protein extracts of cell lysates from day 2; C3, protein extracts of cell lysates from day 3; M1, media harvested day 1; M2, media harvested day 2; M3, media harvested day 3; C3′, C3 incubated at 4 °C overnight after denaturation at 95 °C with dithiothreitol; + , hG-CSF positive control. Numbers indicate the sizes of protein markers in kDa. (**c**) Expression of hG-CSF in the N-rich media. ArM0029B-pSK404 cells harvested on days 3 (C3), 5 (C5), 7 (C7), and 11 (C11) in the same media. Spent media harvested on day 11 (M11) was concentrated 250-fold. M, protein size marker; + , hG-CSF positive control. Numbers indicate the sizes of protein markers in kDa. Red and black arrows indicate hG-CSF monomers and dimers, respectively.

Besides the promoter, the use of an appropriate SP is essential for establishing an efficient protein expression and secretion system. The use of an appropriate SP facilitates the harvesting of proteins from liquid media^38^. To select appropriate SPs for *Chlorella* spp. UTEX 395 and ArM0029B, proteins secreted into media were purified and analyzed using mass spectrometry. Whole-genome sequence contigs of UTEX 395 and ArM0029B obtained from the NCBI (UTEX395-WGS Project accession: LDKB01; ArM0029B-WGS Project accession: JTEE01) were used for gene prediction using the AUGUSTUS software (https://augustus.gobics.de/) with *Chlamydomonas reinhardtii* parameters^39^. From this analysis, a putative cellulase in UTEX 395 and a Ras-related RABF1 in ArM0029B were selected as highly secreted proteins (Supplementary Fig. 5; manuscript in preparation). The sequences predicted using the SignalP program were MAGRITLLLCLCLVAGAAA for the cellulase and MKGALLLLLLALAASAAIA for the Ras-related RABF1 (bold letters in Supplementary Fig. 5). The SP sequences were fused in front of a codon-optimized *hG-CSF* sequence (Fig. 2). The vectors containing the SP of the cellulase were named pSK401 and pSK403 and were controlled by the promoters of *CvNDI1* and *CvNDI2*, respectively. The vectors carrying the SP of the Ras-related RABF were named pSK402 and pSK404 and were driven by the *CvNDI1* and *CvNDI2* promoters, respectively (Fig. 2). Those vectors were introduced into *Chlorella* using the electroporation method described by Kumar et al.^15^.

### Characterization of transgenic *Chlorella* expressing *CvNDI::hG-CSF*

Hygromycin-resistant colonies of UTEX 395 and ArM0029B were obtained within 4 weeks of transformation, using selection agar plates. Individual colonies were transferred to liquid media containing hygromycin and grown for 7 days. Genomic DNA from the liquid culture was subjected to PCR using *hG-CSF* primers (Supplementary Table 2). As shown in Fig. 3a, the incorporation of *hG-CSF* gene into the *Chlorella* spp. was confirmed in the UTEX 395 lines harboring pSK401 or pSK403 (Left panel in Fig. 3a) and the ArM0029B lines harboring pSK402 or pSK404 (Right panel in Fig. 3a). These transformants were successfully maintained for more than 1 year, which indicates that the transformation was stable, as reported previously^15^. To investigate whether the *CvNDI1* and *CvNDI2* promoters were able to induce *hG-CSF* expression under N deficiency conditions, the transgenic *Chlorella* grown in standard nitrogen-rich liquid media were transferred to N-deficient media and cultivated for 3 days. As shown in Fig. 3b and c, all transgenic lines of UTEX 395 and ArM0029B examined expressed *hG-CSF* under N starvation treatment. The amount of transcription of *hG-CSF* increased from day 1 after the induction (Fig. 3b and c). These results indicate that the 1 kb-long *CvNDI* promoters induced the downstream gene in the transgenic *Chlorella* responding to nitrogen deficiency.

### hG-CSF protein was successfully produced in the transgenic *Chlorella*

We next examined whether the *hG-CSF* transcripts induced by the *CvNDI* promoters were translated into the hG-CSF polypeptides in the transgenic *Chlorella*. The production of hG-CSF was examined using the transgenic cell lysates of both UTEX 395 and ArM0029B after 1 day of transfer to the N-deficient media. As shown in Fig. 4a, hG-CSF polypeptides were detected in the transgenic *Chlorella* by western blotting (black arrow). The size of the bands was around 35 kDa in the dimer form, larger than the expected size of 19 kD for the monomer. This observation indicated that the expression system induced the production of hG-CSF polypeptides under N deficiency conditions, although the main form detected was the dimer.

ArM0029B harboring pSK404 was shown to strongly induce the production of hG-CSF (Fig. 4a, Lane 4). Given this result, the production of hG-CSF was further examined using transgenic ArM0029B harboring pSK404 under N deficiency conditions. Cells grown in the N-rich media for 7 days were transferred into N-free media for another 3 days. As shown in Fig. 4b, the production of hG-CSF polypeptides was induced by N deficiency, an observation that matches the accumulation pattern of *hG-CSF* transcripts (Fig. 3b). The polypeptides were most strongly induced at day 3 in the transgenic line, and the hG-CSF produced was successfully secreted into the spent media. The amount of secreted hG-CSF in the pSK404 line was higher than that in the pSK402 line (right panel of Fig. 4b). Overall, the expression vector system controlled by *CvNDI* promoters led to successful production of hG-CSF under conditions of N deficiency, indicating that the system is effective for producing a target protein in *Chlorella*. Commercial hG-CSF synthesized from CHO cells used as positive controls showed a major band at 19 kDa, with a minor band at 35 kDa, identical to the hG-CSF produced in *Chlorella* (right panel of Fig. 4b). The 35 kDa hG-CSF harvested from the transgenic *Chlorella* (Lane C3 of Fig. 4b) was separated into two major bands of 19 and 35 kDa after 4 °C incubation overnight following denaturation at 95 °C with dithiothreitol (Lane C3′ of Fig. 4b), indicating that the 35 kDa molecules might result from dimerization of the 19 kDa monomers.

We further investigated whether hG-CSF expression was induced in N-rich media after additional cultivation in the same media for 11 days without transfer to the N-deficient media. As shown in Fig. 4c, hG-CSF polypeptides were detected in a dimeric form in the 7 day cultivated cell lysates and then decreased in concentration after 11 days of cultivation. In the spent media at 11 days, large amounts of hG-CSF were observed in the form of 35 kDa dimers.

## Discussion

The N starvation condition used in this study was a potential concern with respect to the production of hG-CSF because N is a direct resource for amino acid synthesis. However, we demonstrated that hG-CSF polypeptides were successfully produced up to 3 days after transfer into the N-deficient media. Carbon sources not used for protein synthesis under N depletion conditions are stored as triglycerides and starch in the cells^21^. However, the negative effect of N deficiency on protein synthesis is dependent upon the N level and the duration of treatment^22^^,^ indicating that the flow of carbon from protein synthesis into lipids under N starvation could require more time than we expected. Therefore, the duration—3 days of incubation—used for inducing *hG-CSF* might be optimal for the production of recombinant proteins under N starvation.

*CvNDI* promoters were successfully used for the production of hG-CSF polypeptides in the transgenic *Chlorella* spp. The use of an appropriate promoter is essential for the development of a platform for the high-yield production of recombinant proteins. The *CvNDI1* and *CvNDI2* ORFs were found to encode a urea carboxylase and an AMT, respectively, both of which are involved in N metabolism. Urea is converted into allophanate by CvNDI1 in the first step of urea metabolism^31^. AMT is known to play a vital role in ammonium uptake into a cell, and the transcription of *AMT* genes was found to be strongly induced under N starvation in *Arabidopsis* and *Chlorella*, suggesting that AMT might be an initial sensor of N deficiency^37,40,41^. CvNDI1 and CvNDI2 might be important for the survival of *Chlorella* under N starvation via the utilization of urea as N resource and the enhancing of ammonium transportation in the cell. Therefore, we suggest that the *CvNDI* promoter systems are efficient for the expression of heterologous proteins under N starvation.

Two different SPs identified from UTEX 395 and ArM0029B were used to facilitate the secretion of hG-CSF into the culture medium. The putative cellulase from UTEX 395 appeared to be a polypeptide of 8.4 kDa, and RABF1 from ArM0029B was 17.7 kDa, both rather small polypeptides. Cellulases are known to be secreted from various microorganisms, including fungi and algae^42,43^. RABF1, located at the endosomal membrane, regulates the secretory trafficking pathway in land plants and algae, under stress signaling and senescence^44,45^. Functional analysis would be necessary to characterize those genes. Proteins with small molecular weights (MWs) are secreted more efficiently than larger proteins, and the MW of a secreted protein is one of the major factors related to its secretion efficiency^46^. Strong bands were detected in western blots of the culture media, compared with the band from cell lysates in ArM0029B harboring pSK404. This observation suggests that our secretion system using the RABF1 SP worked efficiently. The cellulase SP from UTEX 395 secreted hG-CSF into the medium as well, when the pSK401 vector was transformed into UTEX 395 (data not shown). Some SPs could not secrete recombinant proteins as efficiently as endogenous proteins^47,48^. Highly expressed proteins with SP could even become cleaved and unfolded^49^. The SP structure could also disrupt the structure of the fused recombinant protein, and those disrupted proteins could induce protease activation, thereby decreasing secretion efficiency^50,51^. Further study into the SPs will be necessary for the establishment of optimal protein secretion systems.

In this study, hG-CSF polypeptides produced in transgenic *Chlorella* were detected with an MW of 35 kDa in both cell lysates and culture medium. This value is higher than the expected size of 19 kDa. This gel shifting in the western blots might be due to either protein aggregation via disulfide bonding or posttranslational modifications such as glycosylation^52,53^. hG-CSF has two disulfide bonds at positions 36–42 and 64–74 and a free cysteine at position 17^54^. The free cysteine was reported to cause dimerization of hG-CSF monomers during protein purification^55,56^. Moreover, recombinant hG-CSF tends to aggregate readily at pH values above 5.0 at elevated temperatures^54,57–59^. Therefore, the pH homogeneity of the culture media during cultivation of *Chlorella* would be important in our culture system. To avoid protein aggregation, further investigation aimed at optimizing protein purification, as well as growth conditions, should be performed. Purified hG-CSF will be used for functional efficacy tests such as growth tests in granulocyte macrophage colonies. Moreover, *O*-glycosylation occurs only at Thr^133^ in native hG-CSF^60^. The glycosylation of recombinant hG-CSF might depend upon the host cells used for hG-CSF production. Lenograstim produced in mammalian cells was found to be glycosylated, whereas filgrastim is produced in *E. coli* in its nonglycosylated form. Thus, hG-CSF synthesized from *Chlorella* might be in the glycosylated form, although *O*-glycosylation patterns were not examined in this study. Protein modification might increase the size of hG-CSF only slightly, because of the small mass of *O*-glycan.

Microalgae have been considered to be an attractive platform for the production of valuable proteins, because of their short life cycle, high biomass, potential for scaling up, and eukaryotic N-glycosylation^45^. *Chlamydomonas* has been an organism of choice for the production of various pharmaceuticals using either constitutive or inducible promoters of *35S*, *Ubi1*, and *HSP70-RBCS2*^45,61–64^. However, its protein production is overall 0.1–5% total soluble protein, which is relatively low compared with the productivity of other eukaryotic organisms^45^. To achieve optimal production of recombinant proteins in *Chlorella*, it will be necessary to identify the copy number and location of transgenes.

In summary, a novel system, including N starvation-inducible promoters, was developed for recombinant protein expression in *Chlorella* spp*.* hG-CSF was successfully produced under N starvation conditions. Functional assays of the protein will be conducted after purification of the protein, and optimization of production will be the next step to produce high yields of hG-CSF from *Chlorella*.

## Materials and methods

### Strain and growth conditions

*Chlorella vulgaris* UTEX 395 and arctic *Chlorella* sp. ArM0029B^28^ were grown in BG11 medium^65^^,^ including 3% glucose at 25 °C under constant illumination (50–60 μmol photons m^−2^ s^−1^) on a rotary shaker (150 rpm) in a multi-spin shaker (Vision Sciences, Korea)^15^. For the transformation of *Chlorella*, we used electroporation, as previously described^15^. Transgenic *Chlorella* colonies were formed on agar plates containing 40 mg/L of hygromycin and were then transferred into BG11 liquid medium containing 3% glucose for further growth. To induce hG-CSF expression, the *Chlorella* transgenic lines were inoculated at 5% (v/v) in 100 ml of new BG11 media and cultivated for 7 days until growth approached stationary phase (OD at 680 nm of 14.0). The cells were harvested by centrifugation at 4,000 rpm for 10 min and then resuspended and cultivated in BG11 media lacking NaNO_3_ for 1 to 3 days. The cells and spent media were harvested for RNA or protein extraction.

### cDNA library construction and massively parallel sequencing

Transcript sequencing of ArM0029B and D3 cultured for 3 days under N-deficient conditions was performed using the Illumina Hiseq2000 platform (Illumina, USA). The detailed procedure was as follows. RNA-Seq paired-end libraries were prepared using Illumina TruSeq RNA Sample Preparation Kits v2 (Illumina, USA). Starting with total RNA, mRNA was purified using poly (A) selection, and then, the RNA was chemically fragmented and converted into single-stranded cDNA using random hexamer priming. Next, the second strand was generated to create double-stranded cDNA. Library construction involved the generation of blunt-end cDNA fragments from ds-cDNA. Then, A bases were added to the blunt ends to prepare them for the ligation of sequencing adapters. After size selection of ligates, the ligated cDNA fragments that contained adapter sequences were enhanced via PCR using adapter-specific primers. The library was quantified using KAPA library quantification kits (Kapa biosystems KK4854) following the manufacturer’s instructions. Each library was loaded onto the Illumina Hiseq2000 platform, and we performed high-throughput sequencing to ensure that each sample met the desired average sequencing depth. Sequence data of quality *Q* greater than 20 were extracted by SolexaQA^66^. Trimming resulted in reads with a mean length of 69 bp across all samples and a minimum length of 25 bp.

### Short read mapping and identification of differentially expressed genes (DEG) and functional annotation

Trimmed reads were mapped to reference transcripts using the Bowtie2 (v2.1.0) software^67^^,^ allowing for alignments with a maximum of two mismatches. The number of mapped clean reads for each transcript was calculated and then normalized using the DESeq package in R^68^. The fold change and number of reads mapped onto reference transcripts were used to identify DEG between each sample. The false discovery rate calculated via DESeq was used to identify the threshold of the p-value in multiple tests and analyses. All correlation analyses and hierarchical clustering were performed using the AMAP library in R^69^. Using Gene Ontology (GO) information and KEGG information (https://www.genome.jp/kegg/) provided by the customer, GO and KEGG analysis of DEG was conducted. The number of genes assigned to each GO term was counted using in-house scripts produced by SEEDERS Co.

### Extraction of nucleic acids and PCR

To extract nucleic acids, *Chlorella* cells were harvested by centrifugation, frozen quickly using liquid N, and then ground using a mixer mill MM 300 (Qiagen, Germany) as described by Kumar et al.^15^. Genomic DNA was extracted from the powder using the cetyl trimethylammonium bromide method^70^. To investigate the presence of transgenes in the hygromycin-resistant cell lines, PCR was performed on genomic DNA using *hG-CSF* primers, which were designed to propagate 351 bp-long transcripts, from 31 to 381 nt of the codon-optimized hG-CSF gene. Total RNA was isolated using Trizol (Invitrogen, USA). After DNase I treatment, the RNA was reverse transcribed at 50 °C for 1 h using TOPscript cDNA Synthesis Kits (Enzynomics, Korea) and subjected to RT-PCR and qPCR using primers for detecting *hG-CSF* transcripts. For RT-PCR, the normalization for quantification was performed by PCR using *Chlorella ubiquitin* (*CvUbi*) and/or *Actin1* (*CvAct1*) gene primers. The number of cycles for RT-PCR was 25 cycles for *CvUBI* and 32 cycles for *hG-CSF*. For qPCR, the comparative threshold cycle method (ΔΔCt) was used (LightCycler 96; Roche, USA). The *CvUbi* gene was used as an internal reference. The primers used in this study are summarized in Supplementary Table 2.

### Vector construction

*hG-CSF* expression vectors were constructed using pJKS136 as a backbone (Fig. 2). The sequences of the promoter regions of the *CvNDI1* and *CvANDI2* genes, spanning the 1 kb-long region upstream of the translation start codon (ATG), including the 5′-UTR, were amplified by PCR, using the genomic DNA of ArM0029B as templates (Supplementary Fig. 4 and Supplementary Table 2). The *CvNDI1* and *CvNDI*2 promoter sequences obtained were inserted into pJKS136 using the restriction enzyme sites *Hin*dIII and *Bam*HI, thereby replacing the *RAmy3D* promoter. Using secretomic data analysis (manuscript in preparation), SPs for protein secretion were extracted from the sequence of the putative cellulase genes from UTEX 395 and Ras-related RABF1 from ArM0029B (Supplementary Fig. 5). SP sequences were fused with the codon-optimized *hG-CSF* sequence (GenScript, USA) by PCR using forward primers containing the SP nucleotide sequence with the 5′ end of the *hG-CSF* (red letters in Supplementary Table 2) and a reverse primer of the 3′ region of the *hG-CSF* sequence. The *hG-CSF* sequence fused with SP was introduced into the region between the *CvNDI* promoters and the *RAmy3D* terminator using the restriction enzymes *Kpn* I and *Bam*HI.

### Immunoblotting

*Chlorella* cultivated in N-deficient media were harvested by centrifugation at 8,000 rpm for 20 min, and then, the spent medium and *Chlorella* were sampled. For extraction of total protein from *Chlorella* cells, 50 mg of cells frozen in liquid N were homogenized in a mixer mill MM300 (Qiagen, Germany) and then resuspended in 1 M PBS buffer (pH 7.4) including 1 × cOmplete™ (Roche, Germany) at 4 °C. The solution was centrifuged at 14,000 rpm for 10 min, and then, the supernatant was transferred into a new tube. To determine the concentration of total proteins in the medium, the spent medium was filtered through a 0.2 μm membrane and was concentrated up to 250 times via 5 kDa size cut-off Viva Flow 200 and Vivaspin 20 in turn (Sartorius, USA). After the cell lysates and total proteins from the medium were separated in 12% NuPAGE gel (Invitrogen, USA), they were transferred onto nitrocellulose membranes using the TurboTransfer system (Bio-Rad, USA). After the membrane was incubated with the polyclonal antibody of hG-CSF (Abcam, USA) for 1 h (1:2000 dilution), it was exposed to anti-rabbit IgG-HRP, which was diluted to 1:5,000 (Abcam, USA). Recombinant hG-CSF synthesized in CHO cells (Abcam, USA) was used as the positive control.

### In silico analysis

BLAST searching using the amino acid sequences of CvNDI1 and CvNDI2 was performed using the NCBI BLAST program (https://blast.ncbi.nlm.nih.gov/). Sequence alignment analysis was conducted using the Clustal Omega program (https://www.ebi.ac.uk/Tools/msa/clustalo/). The hydrophobicity of CvNDI2 was predicted using TMHMM Server v. 2.0 (https://www.cbs.dtu.dk/services/TMHMM-2.0/). The presence of SPs and the location of cleavage sites in proteins were predicted using the SignalP-5.0 program (https://www.cbs.dtu.dk/services/SignalP/).

## Supplementary information

Supplementary file1

Supplementary file2
