# Supplementary material for: The establishment of new protein expression system using N starvation inducible promoters in Chlorella
Source: Sci Rep. 2020 Jul 29;10:12713. doi: 10.1038/s41598-020-69620-9 (PMC7391781; doi:10.1038/s41598-020-69620-9)
Supplement: Supplementary file 1 — Supplementary file1 [file 41598_2020_69620_MOESM1_ESM.pdf]

The establishment of new protein expression system using N starvation inducible promoters in *Chlorella*

Jun-Hye Shin[1], Juyoung Choi[1], Jeongmin Jeon[1], Manu Kumar[1], Juhyeon Lee[1], Won-Joong Jeong[2], Seong-Ryong Kim[1]*
[1]Department of Life Science, Sogang University, Seoul, South Korea
[2]Korea Research Institute of Bioscience and Biotechnology, Daejeon, South Korea

Supplementary Table 1. Genes up-regulated by nitrogen starvation obtained from differential expression (DE) analysis using RNA-seq data.

		**log2FoldChange**
**Genes**	**Functional description**	**29B_vs_N_3d**
scaffold275G00200	amino acid permease (XP_002327371, 2.01E-35)	20.835
scaffold326G00920	urea active transporter-like protein (XP_005645091, 0.00E+00)	11.699
scaffold37G001690	ammonium transporter (XP_002314518, 1.77E-44)	11.639
scaffold164G00210	nitrate transporter NTR (AAK02066, 0.00E+00)	10.301
scaffold326G00270	ammonium transporter (AFV36360, 0.00E+00)	10.096
scaffold326G00910	urea carboxylase (XP_005643671, 0.00E+00)	9.862
scaffold73G00080	GOGAT, glutamate synthase (EMS25597, 0.00E+00)	9.749
scaffold253G00910	acylamide amidohydrolase (WP_019722123, 5.22E-155)	9.394
scaffold11G001150	expressed protein (XP_005843832, 6.69E-07)	8.378
scaffold11G001140	expressed protein (XP_005843832, 4.72E-06)	8.099
scaffold175G00150	late embryogenesis abundant protein homolog (AAA79745, 5.58E-22)	7.732
scaffold301G00550	antifreeze protein (ABR01229, 1.34E-14)	7.407
scaffold305G001070	hypothetical protein CHLNCDRAFT_135268 (XP_005846770, 1.15E-25)	7.26
scaffold10G00510	copper amine oxidase (XP_002959097, 3.69E-179)	7.074
scaffold301G00540	antifreeze protein (ABR01229, 3.39E-13)	6.782
scaffold164G00190	nitrate reductase (ACF22999, 0.00E+00)	6.351
scaffold25G001600	membrane protein containing DUF1538 (EHP30115.1, 6.13E-63)	6.058
scaffold0G00550	(2Fe-2S)-binding protein (WP_006102543, 6.97E-54)	5.909
scaffold80G00050	hypothetical protein CHLNCDRAFT_57598, partial (XP_005848440, 1.37E-165)	5.582
scaffold344G00160	protein serine/threonine kinase, putative, partial (XP_004185680, 1.73E-12)	5.462

Supplementary Table 2. List of primers used in this study.

Primer name	Sequences (5' to 3')	Application
CvUbi-F	GAGTCTTCGGACACCATCGAG	RT-PCR
CvUbi-R	CTTGTCCTGGTTGTACTTGCG	
CvAct1-F	ATGCTTTGCCACATGCCATC	RT-PCR
CvAct1-R	CCGCTGTCTAGCACGATACC	
hG-CSF-F	TCTCTGCCACAGAGCTTCCT	gDNA PCR & RT-PCR
hG-CSF-R	CATCTGCTGCCAGATTGTTG	
CvNDI1-F	TGGTGTTCATTGCCCTGTGT	RT-PCR
CvNDI1-R	GGAGGTCCATAGCAACCGAG	
CvNDI2-F	CGTGGTACCTCACAGGCTTT	RT-PCR
CvNDI2-R	GCGACCACAGGGTAGACAAA	
scaffold73G00080-F	CTGTCACACGAGGTCAGCAA	RT-PCR
scaffold73G00080-R	GTACCGCCGGTCATGTACTC	
scaffold37G001690-F	GCCAAGCCCAGAATCAGTGT	RT-PCR
scaffold37G001690-R	TTGCATCTGCGTCTTTGTCG	
*Bam*H I_UTEX395 SP_CSF-F	CGCGGATCCATGGCCGGCCGCATCACCCTGCTGCTGTGCCTG TGCCTGGTGGCCGGCGCCGCCGCCATGGCAGCCCTCTC	Cloning
Kpn I_CSF-R	GCCGGTACCGGGCTGTGCCAGG	Cloning
*Bam*H I_29B SP_CSF-F	CGCGGATCCATGAAGGGCGCCCTGCTGCTGCTGCTGCTGGC CCTGGCCGCCAGCGCCGCCATCGCCCGCGACATGGCAGCCCT CTC	Cloning

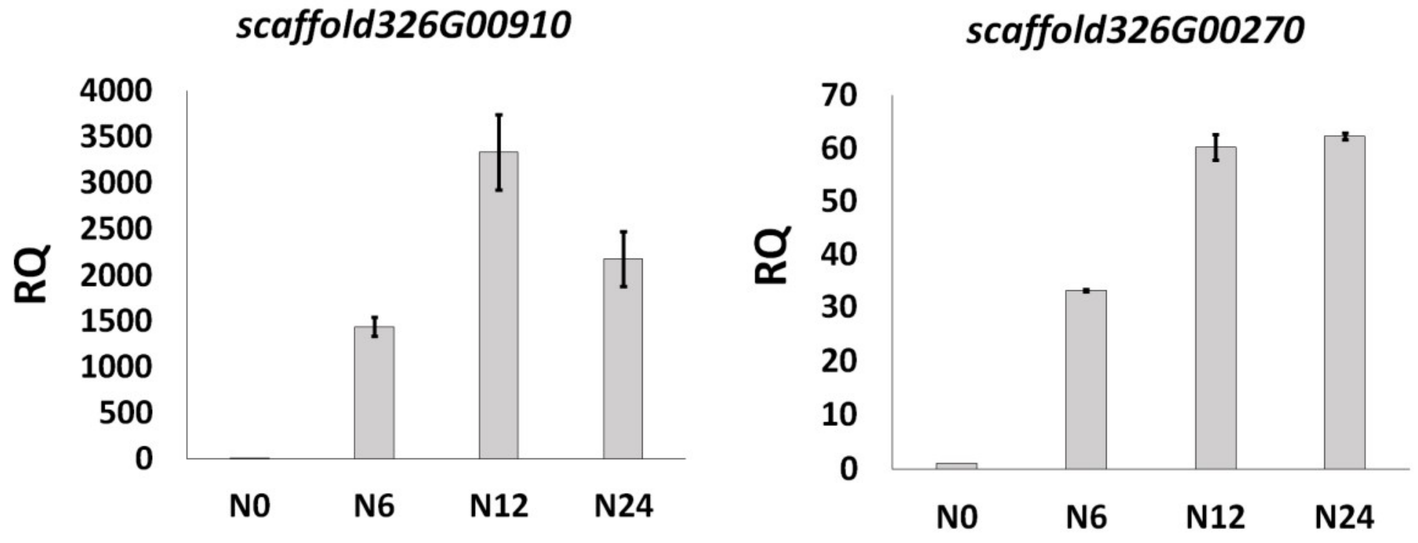

Supplementary Figure 1. RT-qPCR analysis of two candidate genes (*Scaffold326G00910* and *Scaffold326G 00270*) detected in N deprived growth conditions after 0, 6, 12, and 24 h which are represented by N0, N6, N12 and N24.RQ indicates relative quantity, and error bars represent standard error of three replicates.

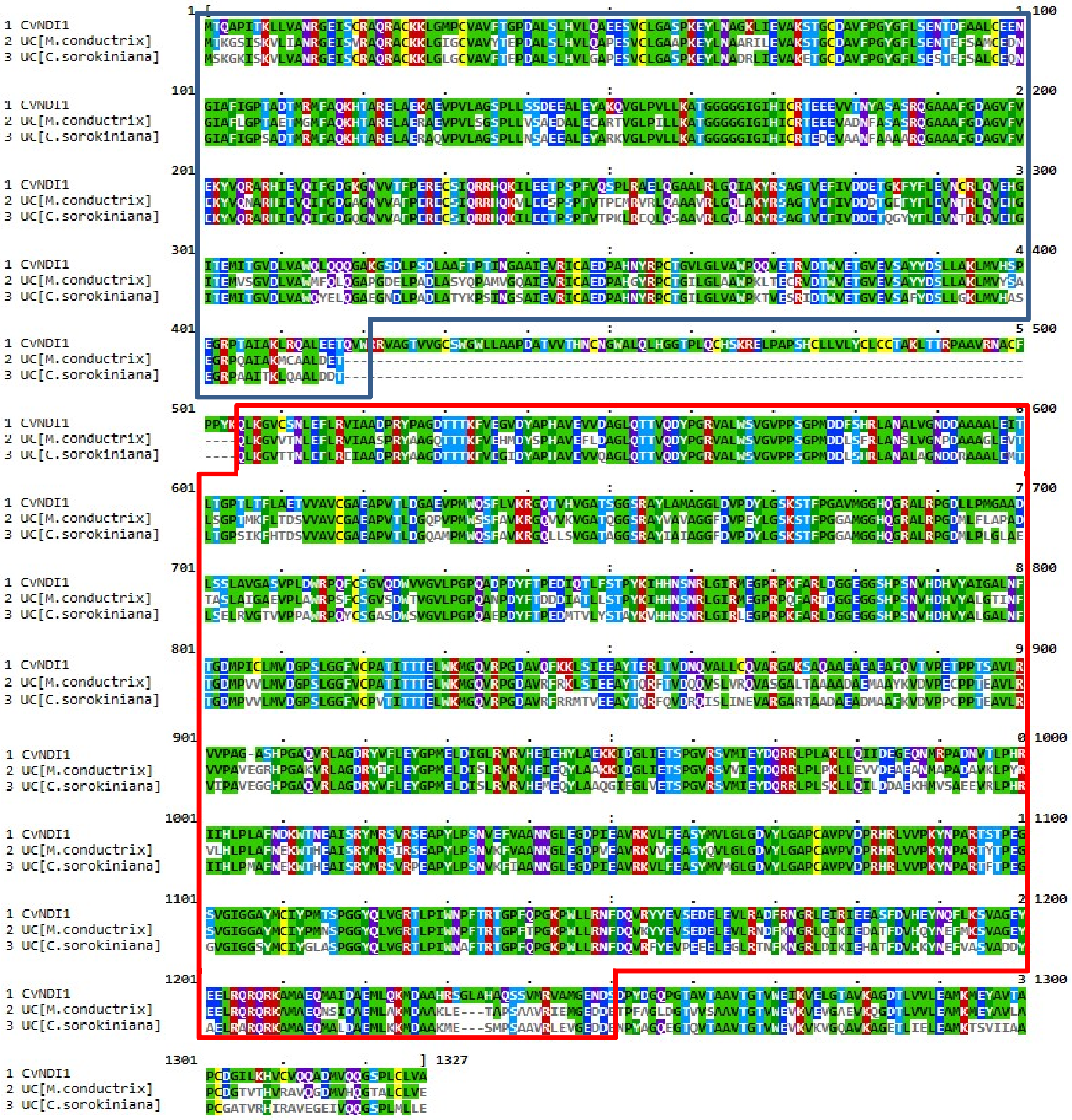

Supplementary Figure 2. Amino acid sequence alignment of CvNDI1. The amino acid sequence predicted based on *CvNDI1* gene sequence was aligned along with urea carboxylases (UC) of other microalgae. Clustal omega program (https://www.ebi.ac.uk/ Tools/msa /clustalo/) was used for the alignment. Blue and red boxes mean biotin carboxylation domain and carboxyltransferase domain respectively.

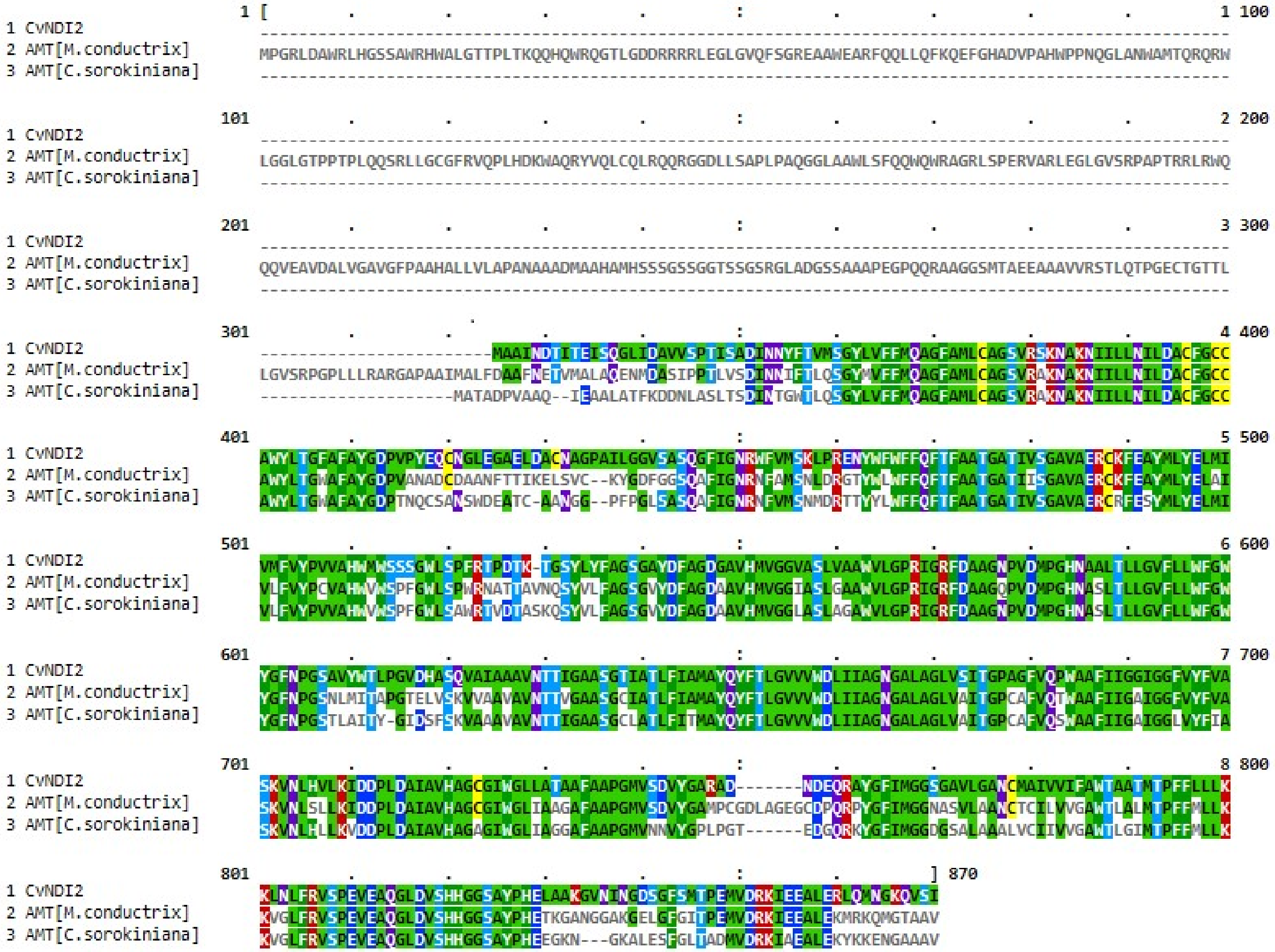

Supplementary Figure 3. (a) Amino acid sequence alignment of CvNDI2. The amino acid sequence predicted based on CvNDI2 gene sequence were aligned along with ammonium transporters (AMT) of other microalgae. Clustal omega program (https://www.ebi.ac.uk/Tools/msa /clustalo/) was used for the alignment.

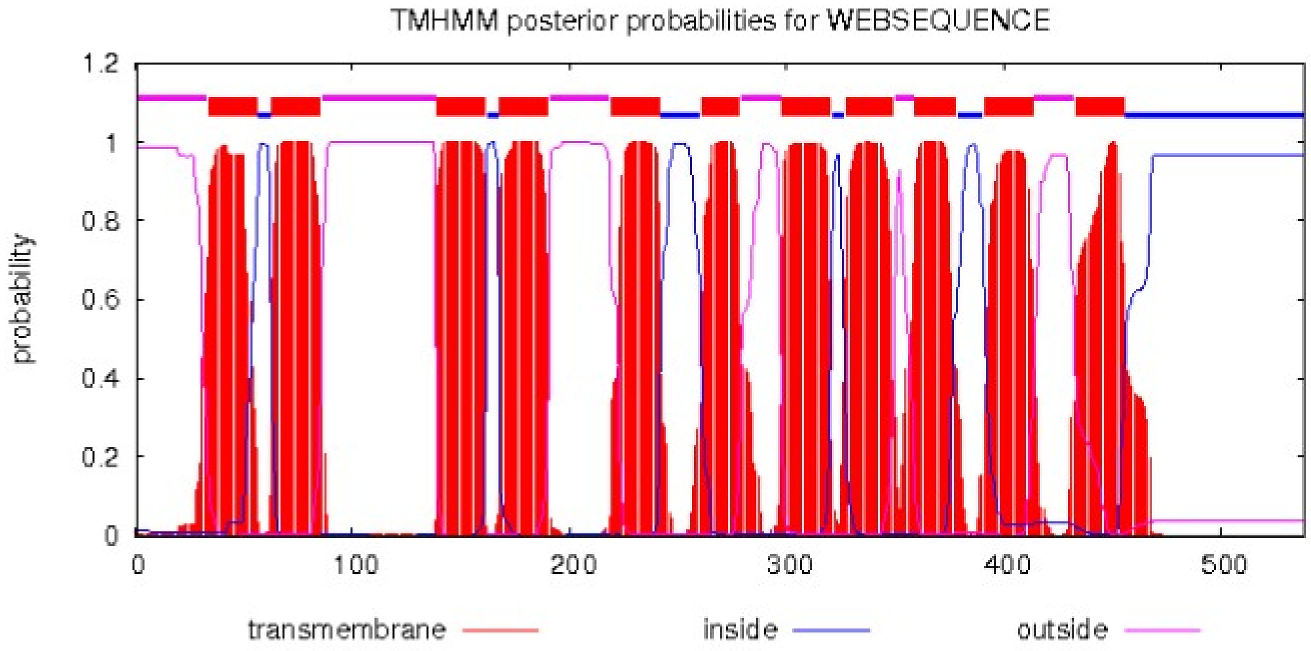

Supplementary Figure 3. (b) The hydropathy profile of CvNDI2. Red color indicates the transmembrane domain, and the inside and outside regions are in respectively blue and cyan.

>*CvNDI1* promoter

```
CGCGGTTGGCCACCAGCAGCTTGGTGATGGGAGCCTGGGTCATGGCGACTGGGTCTGCAG
GGCAACGCGAGCAAAAAGGGAGCGGTTGAGCAGGCAGTGCCGCCGCCAGCTGCCGGGAGC
GCAGGCAAGCGTTGGCCCGCATTGCCGGTGGCGCATTCTGAAATGCCGGGTTGCGTGCGT
GTGTGCTCACAACACGCTGACAGCGAAAACCGACGCAGAACCCCACACAGGCCGGGGTGC
CCCCTCCCGCCCTTGCTGCGTCTGCCGGCCGCATCAGTTTATCATTGATGTCACGATGCA
TGCATTTGCTATGAGGCCCTGCTCCCAAAACTAGGGCTCTGCCCCGTGCATCACCTGCGC
AGTCCCTCTCGCGATGCGCAGGCGCACCACAATGGTGCCAATGCTGCAGAGAAGATACAA
CCAAGAAGATACAACCAAGAGGAGGAGAAGCGGCCATCGTTGGGTCCATCACCGACTGCC
AGCTGGCTCCTCCCGCCGCAATCCGCAACCCTTGCGCGGCACATCTGGAGACAGCTGGCA
GCTGGCCTGTGCTCAACACGCCCTTTACCTATCCTCTTCGCCGCCACAATCAGTGTCCCA
CATTTGTGAGTGCAAGCCGAGCCCAACCGGATTTCATTTACGCATGACCGCCTACACGCC
TGCGGTTGCGGCGCTGCGCTGCCGAGTGCGTCACTCGTTTTTACAGCGACCCACACCACG
GCACTCTGTAGGGAGCATCAACTGAGCCCACCGTCGTCCGATCCCGTCAACATCACCTGG
GGAAGCTGCTGCTGCCTGAAAAGGATGTGACCCTGGTACCAACTTTTCTGCCCCTCTGAC
GGGGCGGTGGTCTCTCATGCATTGGGTTCACCCATATATTGCGCGGGCACACACTCCGCC
CCCCATTCTTTTCAACTCTCACTCCTCATCCCCCAGGCACGTTGCCACCGCTCAGCAGGG
CCGGGGAGCTCCCATCACCTTGGCGAA
```

>*CvNDI2* promoter

```
GGGCTCTGGGTCCACCTGAAAAGTGGGTCGCCTTTGTGCTTGTTCATCAAAGTTCTTTCA
AGGGCAGATCGGAAGGCGTAACCTCTGCTAGCCATCTGCCTGCACCGCCTCCGCCACCAC
CTCTACCCACTGGCCGCCATGTCTTCGAGACGGCATCATGATGGAATGGAATGTTACATG
CATCAGTGATGCAAACAAGCCTACACAACCCATTATGTGCGCTGCGCTGATCAAGCTGGA
TCCCCGTCCCCCTCGCACCCCAGCACACCGCCAAGCAGTCCGCGATCCACCAATTCCTGC
ACCAAGCCTCTGGGCAGCCTGTGGGCAGCCAGACCTTTGTACCACAGCACCGCAGCGCCA
ACCTCGGCTGCCGACAGCCTTTGAGCGGCACTTGGCCCCGCCCTTGAGGCACTTTCGCCG
CTGGCGTTGCTGCTGCTGCTGGTGCTGTTTCTGGTGCTGCTGCCCTCCCCGGGCTTGGCC
CTGCCGCCAGTTCTCGACACCGCTGCGGCAGCCATGGCAGCAGCCCTGCTCGCAGGTGCC
AGCACTAGGAGTGCATAGGCAGCTTGCAGCCCCAGGCAGTCGATTACGGTATCTAACGCT
GCAAGCCGTCGTTGATGGCAACGACTCAGCTGCAGCTGCTGGTTGCGAGCCATGCCCAGA
GCCTCGAGCTTGGCAGCGCTGCAGGCAACAGGCAGGGCGCGGCACAGTTGCTGACATCAG
CACAGCAGCGCAACAGCGTCATGTTGCACTGCAGCAACTCCTCAACACTCGACTGCACAG
CTGGCCACAGCCGTGCACGCTTTGCCACATGCCGCACAGCACAACACACATACCGGTCTG
GCGCCAGCCTGCCCCGCTGCCACTGGTGCAGCTGGAACGAGGCCCAGGCACGCAAGCCGT
CGCCGGCAGGGAGAGGTGCGCTCAGCAGGTCCCCATCACGCTGCTGGTGCAGCTCACAGA
GCTGAGTGTAGCGGCGGTCCCACCGGCCCTCCCTTGGGT
```

Supplementary Figure 4. Promoter sequences of *CvNDI1* and *CvNDI2* genes.

>Cellulase (UTEX 395)
MAGRITLLLCLCLVAGAAAAPIDAATACSKMPATWKPVETVPDEVSWAVYSAAYDRY
AGSGLDIDWTSYYCAEPTYSYDGCYAVSSTPLLARLPAR

>Ras-related RABF1 (ArM0029B)
MKGALLLLLLALAASAAIARDLETWGHHGKKKVKVVPVVVKPKPVVVVKPKPVVVVK
PKPVVVVKPKPAVVVVAKPEQKYPVCDGAVLASCCYLPIQYDYYGKAASTCKIVDKVA
VPIHPWYPKKMVAKEVVKHLYLASYPPPVYTDGASFACVCPTACEVPSPCSPYPCTVH
MPDCGGYGK

Supplementary Figure 5. Amino acid sequences of cellulase and Ras-related RABF1. Bold letters indicate predicted signal peptide sequences.
