# Supplementary material for: The establishment of new protein expression system using N starvation inducible promoters in Chlorella
Source: Sci Rep. 2020 Jul 29;10:12713. doi: 10.1038/s41598-020-69620-9 (PMC7391781; doi:10.1038/s41598-020-69620-9)
Supplement: Supplementary file 2 — Supplementary file2 [file 41598_2020_69620_MOESM2_ESM.pdf]

The establishment of new protein expression system using N starvation inducible promoters in *Chlorella*

Jun-Hye Shin[1], Juyoung Choi[1], Jeongmin Jeon[1], Manu Kumar[1], Juhyeon Lee[1], Won-Joong Jeong[2], Seong-Ryong Kim[1]*
[1]Department of Life Science, Sogang University, Seoul, South Korea
[2]Korea Research Institute of Bioscience and Biotechnology, Daejeon, South Korea

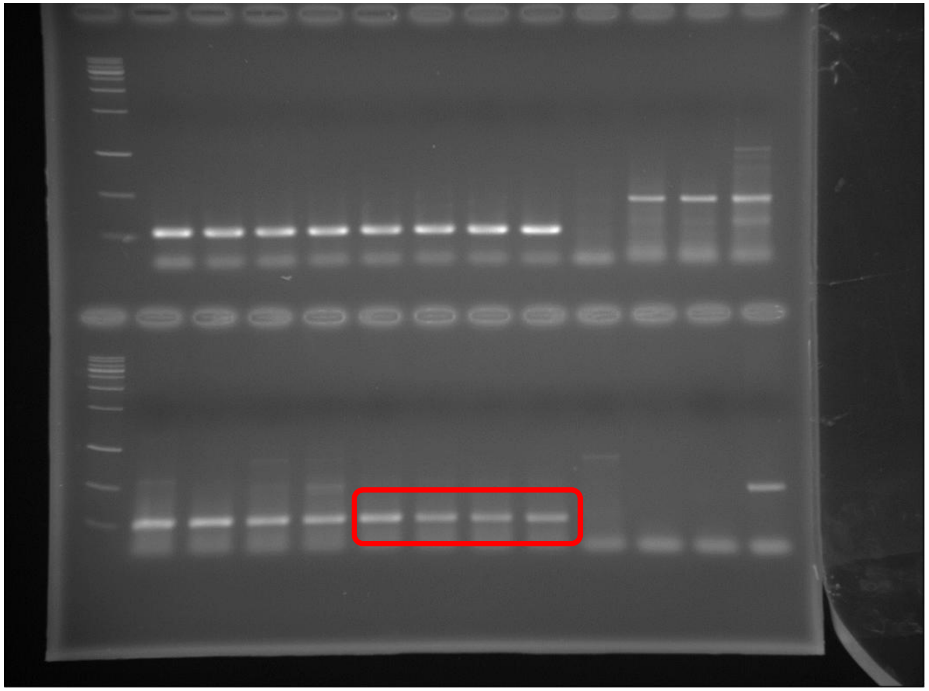

Fig 1_CvAct1

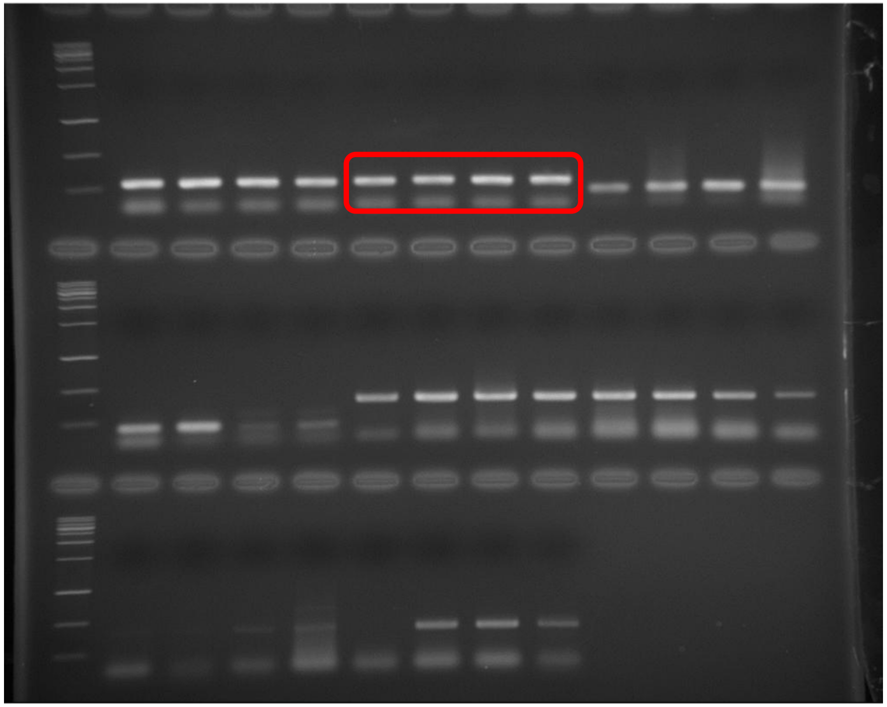

Fig 1_C.Ubi

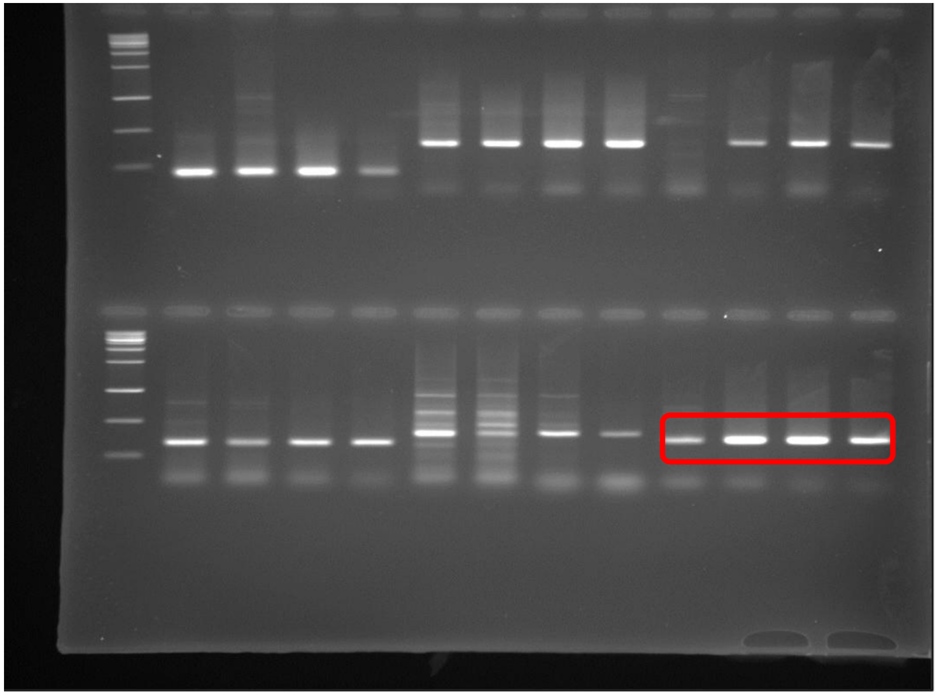

Fig 1_Scaffold326G00910

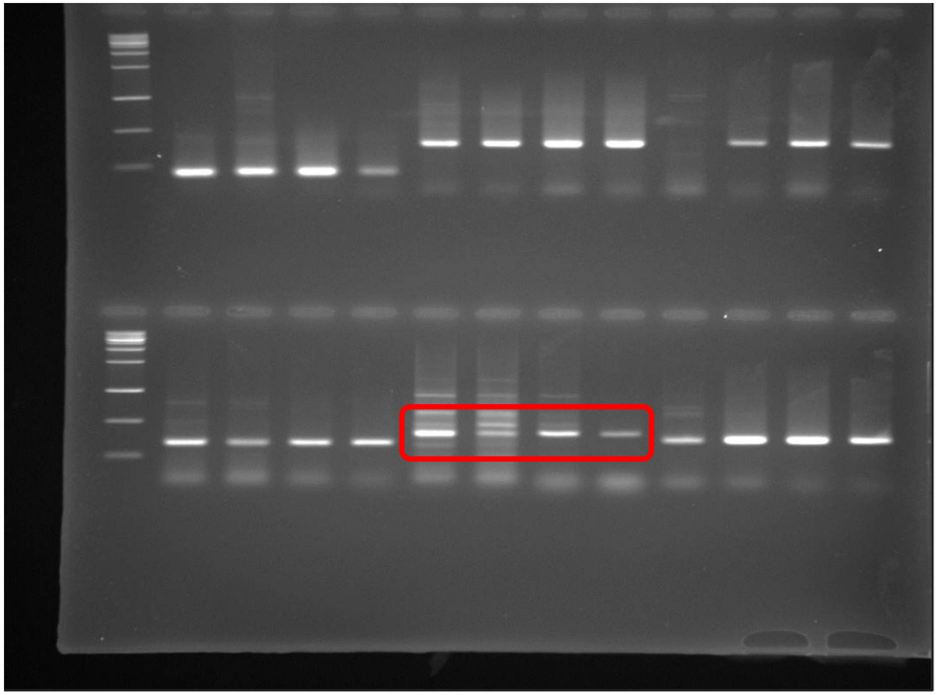

Fig 1_Scaffold73G00080

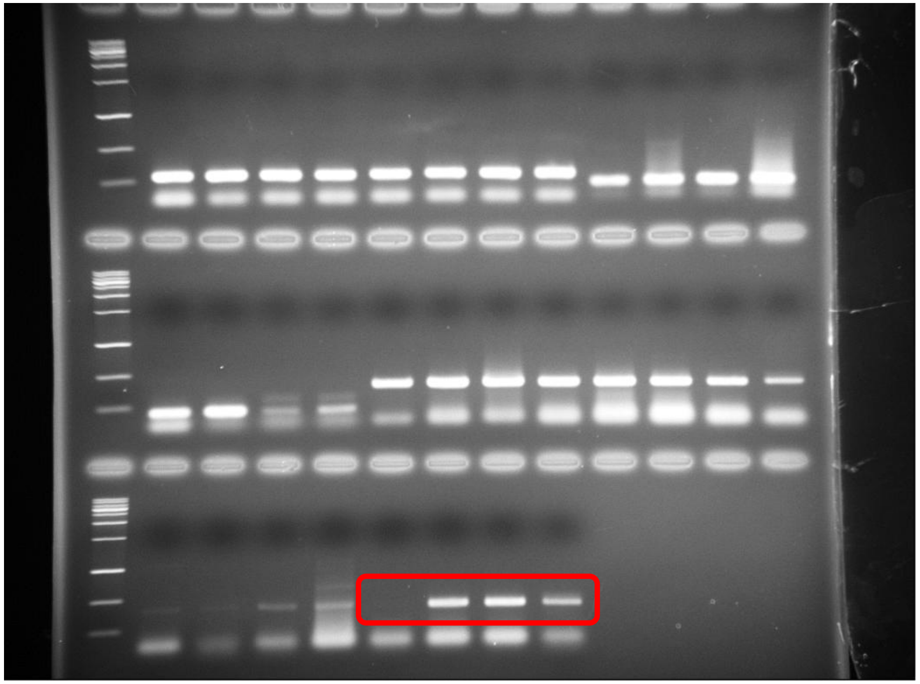

Fig 1_Scaffold326G00270

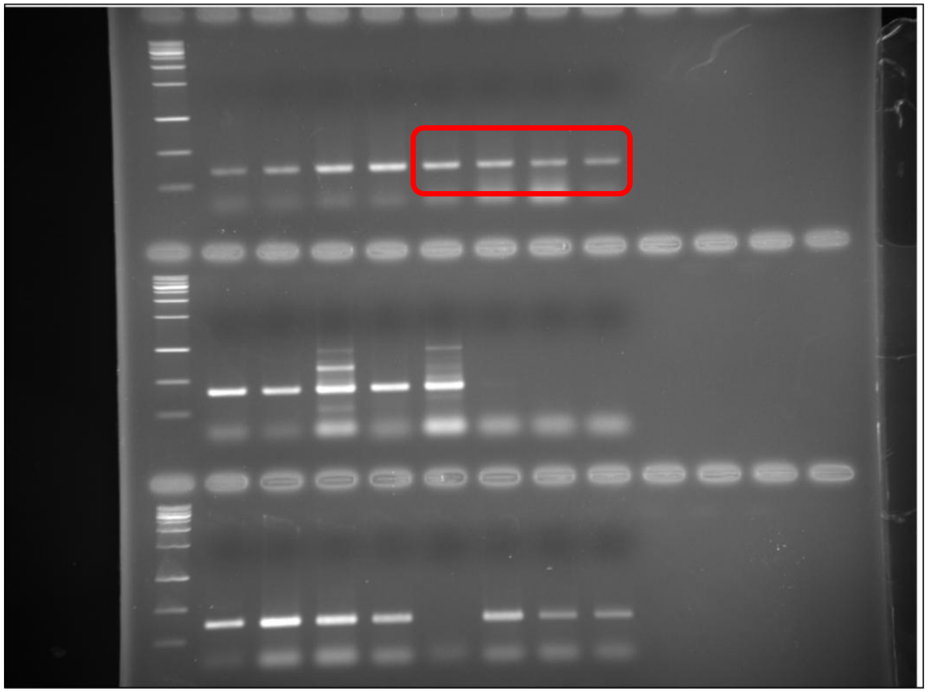

Fig 1_Scaffold37G001690

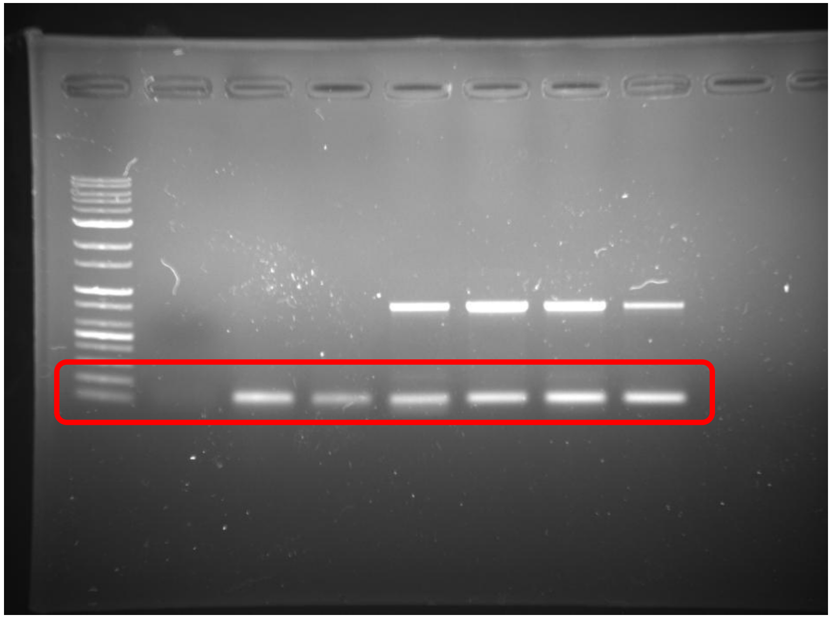

Fig 3a_UTEX395 PCR

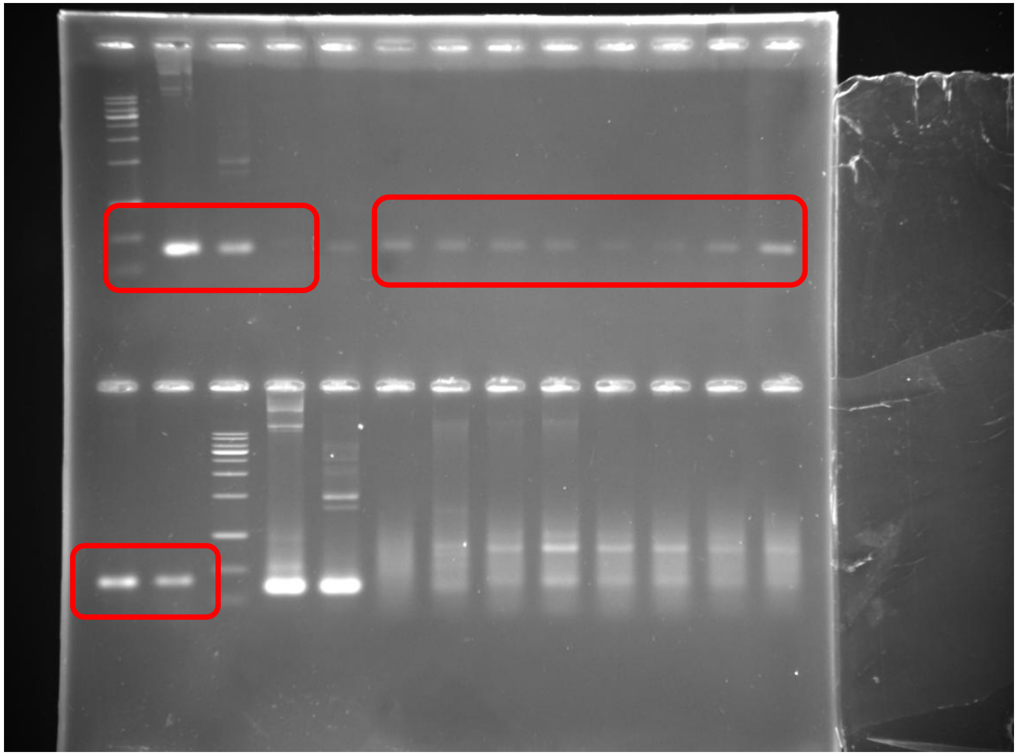

Fig 3a_ArM0029B PCR

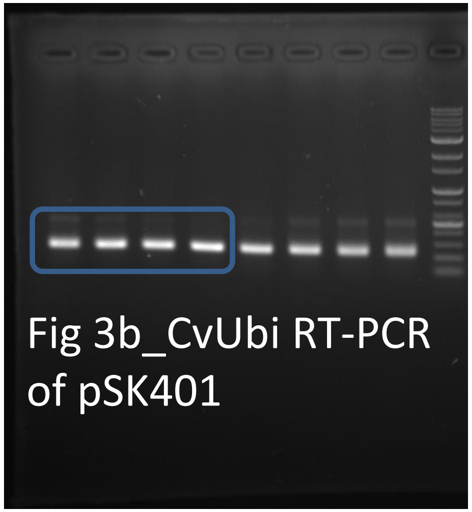
Fig 3b_CvUbi RT-PCR of pSK401

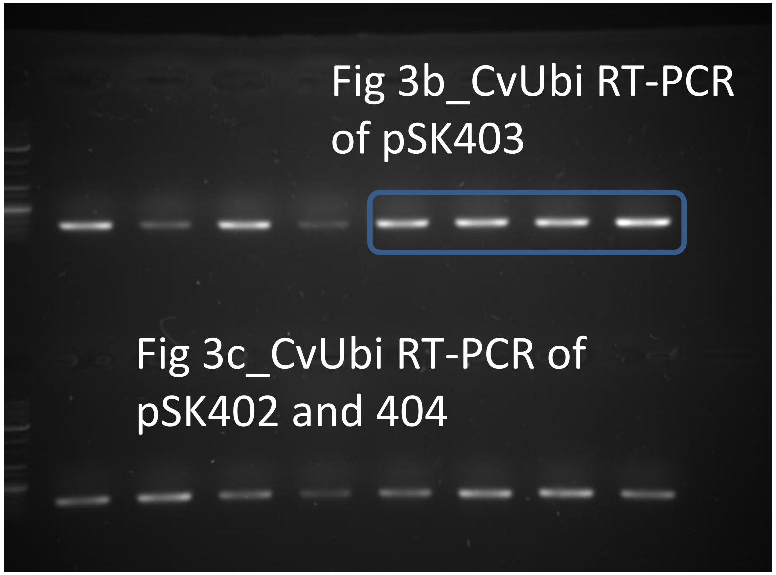
Fig 3b_CvUbi RT-PCR of pSK403
Fig 3c_CvUbi RT-PCR of pSK402 and 404

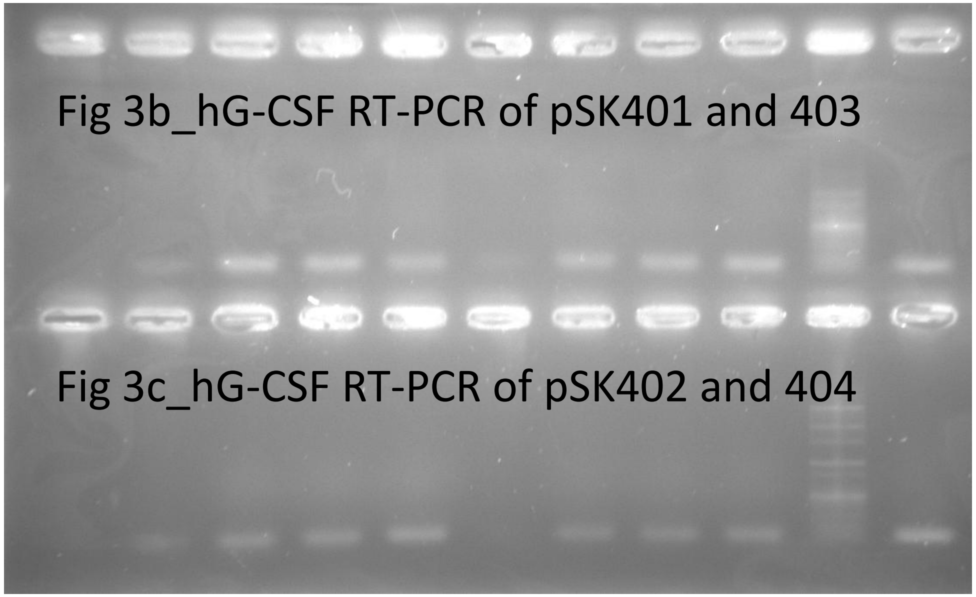
Fig 3b_hG-CSF RT-PCR of pSK401 and 403
Fig 3c_hG-CSF RT-PCR of pSK402 and 404

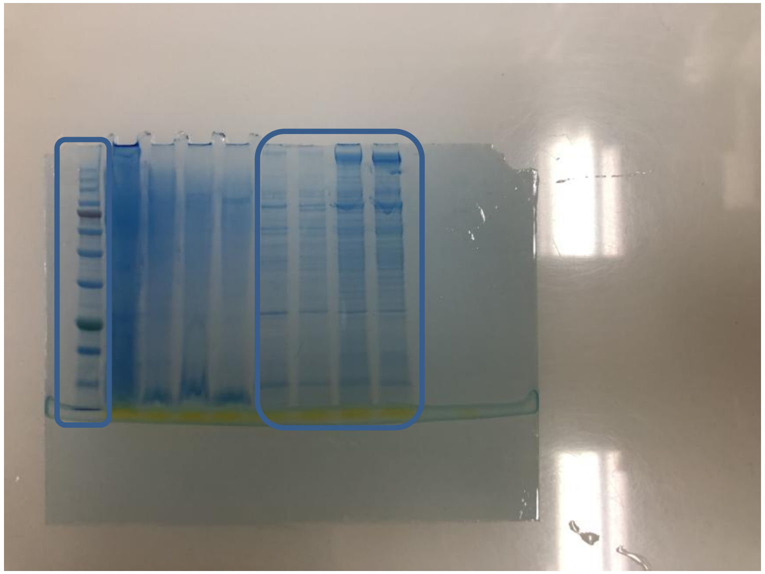

Fig 4a_left panel

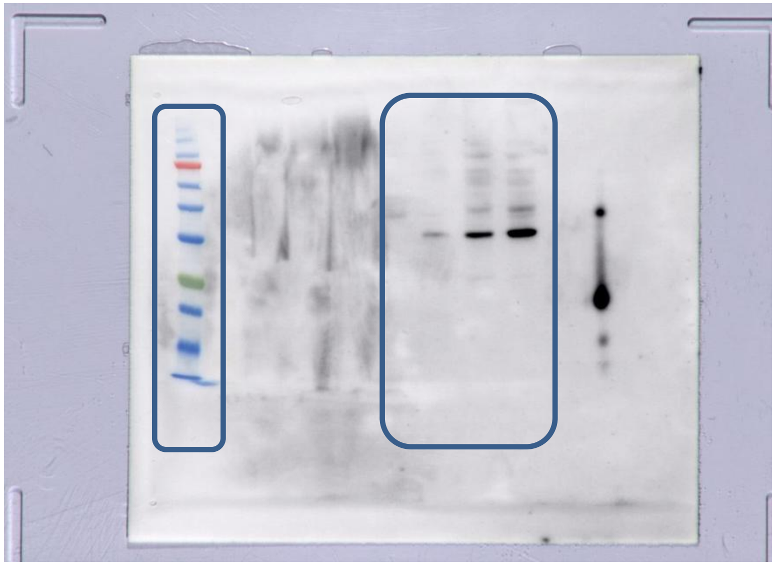

Fig 4a_right panel

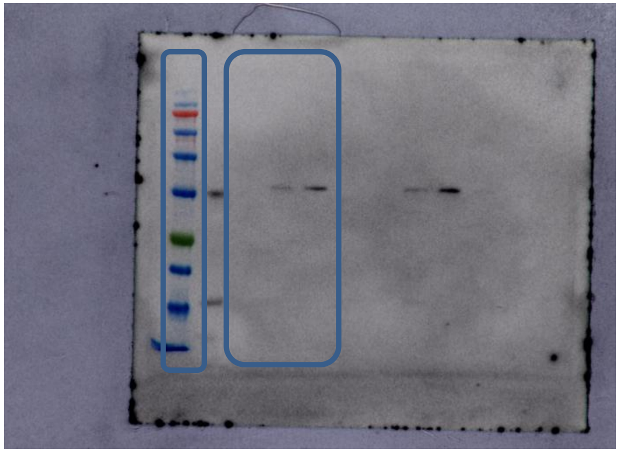

Fig 4b_left panel

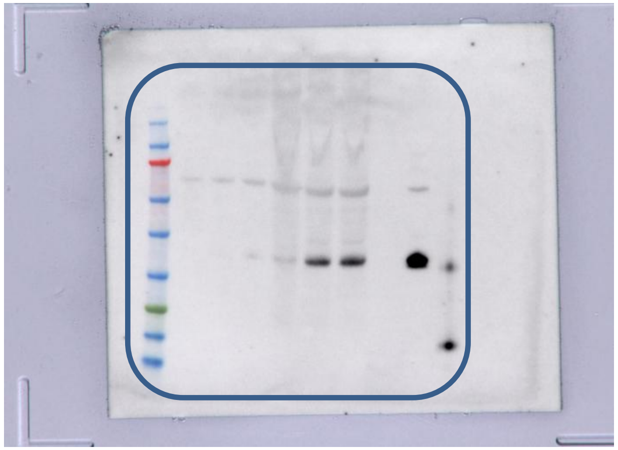

Fig 4b_middle panel

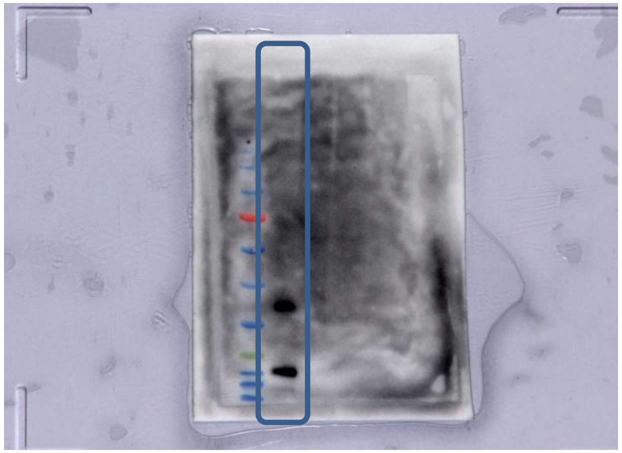

Fig 4b_C3’ in middle panel

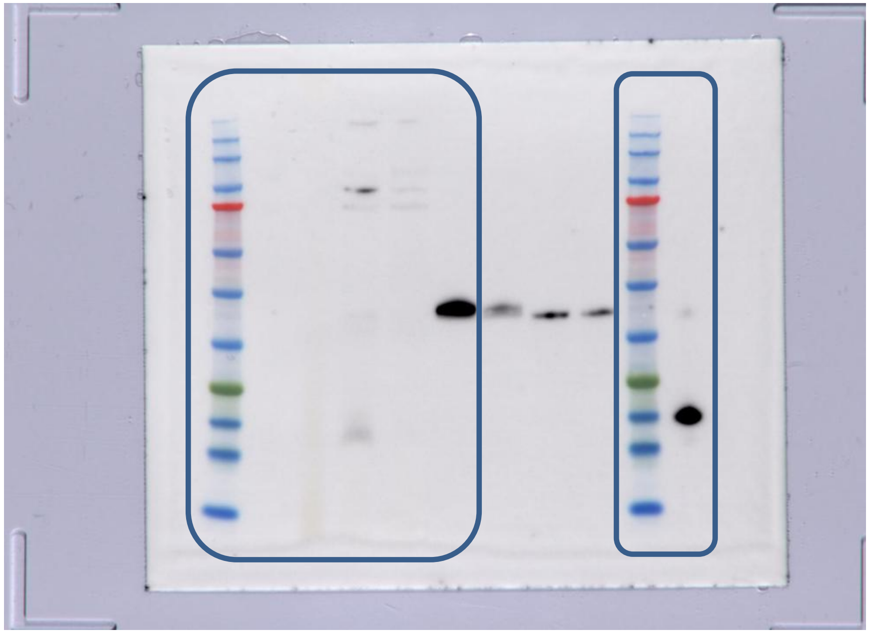

Fig 4c
